# The Role of Fundamental Movement Skills and Health-Related Fitness on Physical Activity During Guided Active Play for 8- to 10-Year-Old Children

**DOI:** 10.3390/children12060805

**Published:** 2025-06-19

**Authors:** Glory Madu, Victoria Kwong, Dusan Calic, Taylor Cleworth, Angelo Belcastro

**Affiliations:** 1 School of Kinesiology and Health Science, Faculty of Health, York University, Toronto, ON M3J 1P3, Canada; glory95@my.yorku.ca (G.M.); vickykwong88@gmail.com (V.K.); calic.dusan.pt@gmail.com (D.C.); tclewort@yorku.ca (T.C.); 2 Muscle Health Research Centre, Faculty of Health, York University, Toronto, ON M3J 1P3, Canada

**Keywords:** children, middle childhood, motor skill proficiency, structured observational study

## Abstract

**Background:** Active play has been proposed to complement school-based physical activity (PA) and promote increased movement-related activities relevant for the development of motor competence. Guided active play (GAP) paired with cooperative games provides sufficient moderate-to-vigorous physical activity (MVPA) to improve motor competence for younger children. Whether guided active play exhibits physical activity outputs that are related to motor competence is uncertain. This study assessed the strength of relationships between play-based physical activity and movement skills by comparing linear regression and chi-square analyses. **Methods:** Forty-two children (Mage = 8.8 ± 0.8 years) participated in a community center program. PA was measured via accelerometry for GAP, alongside assessments of anthropometrics, fitness (leg power, strength, VO_2_max), and FMS (Test of Gross Motor Development-2). Multiple linear regression analysis examined reciprocal relationships. Chi-square and cross-tabulations analyzed categorical variables based on lab percentiles (low < 33%, high > 66%) for PA energy expenditure (PAEE), intensity (MVPA), FMS, and fitness. **Results:** GAP MVPA and object control skills (OC) showed positive reciprocal pathways (β = 0.308, β = 0.394; *p* ≤ 0.05). VO_2_max predicted MVPA (β = 0.408; *p* < 0.01), with leg power related to PAEE (β = 0.456; *p* ≤ 0.01). Chi-square analysis revealed significant associations between high OC skills and high PAEE (X^2^ = 15.12, *p* ≤ 0.05), and high individual average scores of OC with high MVPA (X^2^ = 11.90, *p* < 0.05. The high performance of AP and LP was associated with MVPA and PAEE, respectively. **Conclusions:** Findings support a positive feedback loop between MVPA and OC skills for GAP. GAP is an effective strategy for program interventions for children 8 to 10-year old.

## 1. Introduction

During childhood development, both physical activity (PA) and fundamental movement skills (FMS) are important factors for improving physical health, cognitive development, and social-emotional well-being [1,2,3]. FMS, including locomotor (LOC) skills, such as running, jumping, and object control (OC) skills (i.e., throwing and catching), are considered the building blocks for more complex movements and are essential for participating in everyday tasks, sports, and physical activities [4]. To better understand FMS changes, a conceptual model by Stodden and his colleagues proposed reciprocal bidirectional and developmentally dynamic relationships among FMS levels; PA outputs, including time spent in PA and moderate-to-vigorous PA (MVPA); and health-related fitness components (HRF), such as cardiorespiratory fitness, muscle power, and strength [5]. It is hypothesized that encouraging PA participation and enhancing HRF is important for improving LOC and OC skills during early childhood [5]. During middle to late childhood, the gains in FMS proficiency and HRF are hypothesized to contribute to increased PA participation to promote health-related and sport-related outcomes [5].

Review reports have suggested that the hypothesized relationships between PA and FMS are more complicated than previously proposed [6,7]. For example, an investigation into the reciprocal relationships between FMS and school-based PA during middle childhood showed non-significant direct effects for both directions [8]. Another study focusing on identifying relationships between motor competence and school-based PA for children during middle childhood showed weak correlation coefficients ranging from r = 0.14 to 0.27 [9]. These results support the view that the PA effect on motor competence is limited and that the nature/quality of the school-based PA may have an influence on the relationships between PA and motor competence, in particular, the observations that school-based PA with a range of PA domains might contribute to the indeterminate/weak associations between FMS and PA [10,11]. For example, children aged 8 to 10 years spend an average of 32 min (7.2%) of the school day (440 min) in MVPA distributed over physical education classes (16 min), recess (13 min), and lunch break (3 min) [11]. Additionally, children’s average daily MVPA is higher during weekdays, compared to weekend days, which is consistent across FMS levels and sex [12].

While school-based PA provides valuable insights into children’s activity habits, particularly in sedentary and light-intensity activities, they do not effectively capture higher-intensity and/or skilled-related activities, especially for girls in the middle to late childhood stage [11,13]. As a result, active play has been proposed to replace skilled-based physical education sessions to provide more autonomy and enjoyment for younger children to be physically active [14]. Furthermore, guided active play (GAP) characterized by self-paced, freely chosen activities and minimal structure and instructions may provide more varied PA opportunities that are associated with more FMS [10,15,16]. It has been proposed that if children’s PA activities include selected movement and performance skills, associations for PA and FMS may improve [17]. Moreover, understanding the role of cardiorespiratory and muscular fitness variables with GAP PA and/or FMS levels could prove important for promoting improvements in PA outputs and FMS. Whether active play, specifically GAP, could address the challenges identified with school-based PA and play a role in the relationships among PA, FMS, and HRF for older children is uncertain.

Guided active play promotes varied PA and FMS opportunities by using several movement and performance skills; however, the use of cooperative (social) games has been reported to align with important PA behaviors for children [14,15,16]. Specifically, the selection of cooperative games can align with movements associated with LOC and/or OC subtype categories [16]. Selecting movements that may promote one and/or a combination of LOC and OC skills presents a challenge when interpreting FMS scores. For example, when computing LOC and OC sum scores, the TGMD-2 assumes an equal weighting for each LOC and OC skill; however, unequal weightings have been reported for the Test of Gross Motor Development-2 (TGMD-2) outputs [18]. The unequal weighting of individual LOC and OC skills may bias their relationships with PA and/or HRF. To mitigate the challenge of unequal factor (skill) weightings, the use of relevant cut-off thresholds can be applied to create classifications composed exclusively of high variables [19]. The application of cut-off thresholds to classify high FMS skill categories with HRF in youth has been reported [20]. To date, no study has compared reciprocal bidirectional relationships between PA and FMS with categorical associations between high LOC and OC skills and high GAP PA outputs in 8- to 10-year-old boys and girls. In addition, comparing the effects of HRF components (cardiorespiratory and muscular fitness) on PA and FMS with the associations observed between categories for HRF and LOC, OC, and PA might prove relevant when describing intervention programs for enhancing PA participation.

This study investigates the relationships between FMS and PA during guided active play for 8- to 10-year-old children. The study aims to observe the PA responses for energy expenditures (PAEE) and metabolic equivalent intensities (MVPA) with HRF, LOC, and OC skills during 1 h of children’s guided active play employing cooperative games. Finally, associations will be determined for guided active play responses by comparing high cut-off classifications between PAEE, MVPA, and HRF with high LOC and OC. The objectives are to:

1. determine the strength and preferred direction of reciprocal relationships between LOC and OC skills and PAEE and MVPA for 1 h guided active play using multiple linear regression adjusted for sex and body mass index.
2. identify the responses for HRF components, including aerobic power (AP), leg power (LP), and strength (STR), on the reciprocal relationships between LOC and OC, with PAEE and MVPA adjusted for sex and body mass index.
3. investigate categorical associations with cut-off thresholds between high-performing groups in LOC and OC skills, high individual average scores in locomotor (LIA) and object control (OIA) skills, and high HRF classifications with high-performing groups in PAEE and MVPA from 1-h guided active play sessions.

## 2. Materials and Methods

### 2.1. Participants

A convenient sample of 42 children out of 57 registered children, aged 8–10 years (mean age 8.8 ± 0.8 years), registered in two community-based summer day camp programs, were recruited. The center was in a large urban community identified as an underserved community with lower socio-economic status compared to other neighborhoods in the city. Children were included in the study if they provided an informed consent form and completed the physical activity readiness questionnaire. Seven (*n* = 7) children were excluded from the study for incomplete consent information; eight (*n* = 8) children were excluded for incomplete data associated with absences on test days.

### 2.2. Study Design

The study was a quasi-experimental repeated measures design in a community-based summer day camp that spanned 6 weeks, five days a week from 8:30 a.m. to 3:30 p.m. Guided active play PA sessions were scheduled for 1 h per day over 4 days each week, excluding weekends. At the start and end of the camp, assessments and measurements were conducted in a gymnasium for FMS and HRF. During weeks 1 and 6, all children participated in guided active play sessions using cooperative games such as Mr. Wolf, Octopus, Crocodile, etc. [16]. The sessions were prepared to increase opportunities for children to participate in self-paced activities and enjoy the experience [16,21]. The guided active play sessions, FMS, and HRF assessments were led by the principal investigators and experienced PA leaders (*n* = 12), with additional support from graduate students and/or undergraduate kinesiology majors (*n* = 6) who completed 3–5 training workshops. This structure ensured a consistent delivery and contributed to the reliability and validity of the sessions and assessments.

### 2.3. Anthropometric Measurement/Procedures and Health-Related Fitness

Standing height and body mass were measured using a stadiometer and electronic scale. Body mass index (BMI) was calculated according to previously established protocols [22,23]. For HRF components, aerobic power (AP), expressed as VO_2_max mLO2·kg·min^−1^, was estimated from a 20 m shuttle run [24]. Leg power (LP), expressed in Watts, was derived from standing vertical jump height and body mass [25]. Strength (STR) was measured using a hand grip dynamometer and expressed in kilograms (kg). Grip strength was measured in two trials per hand, performed alternately. The mean score for each hand was then combined to obtain the overall grip strength STR score [26]. The coefficient of variability for all measures was acceptable at +/−3%. Finally, the interclass correlation coefficient (ICC) for TGMD-2 was calculated, achieving a value of 0.97 with a 95% confidence interval ranging from 0.94 to 0.99 across all assessors.

### 2.4. Fundamental Movement Skills

The Test of Gross Motor Development-2 (TGMD-2) was used to assess LOC (run, hop, leap, horizontal jump, slide, gallop) and OC (striking, kicking, dribbling, catching, throwing, rolling) subtest scores as outlined in the TGMD-2 Examiners Manual [27]. Performance criteria for each skill were assessed during two test trials. A score of 1 was given if a criterion was met, and 0 if not. Mastery was defined as meeting all criteria for a skill, and proficiency as meeting all but one [27]. All assessors’ scores were validated against those of an expert (PhD, 10 years of experience) to ensure content validity.

### 2.5. Physical Activity

Children’s physical activity (PA) levels were quantified and characterized using right hip-worn accelerometers (ActiGraph GT3X+) (ActiGraph, Pensacola, FL, USA) and direct observation during 55 min of the guided active play session. The sampling interval was set at 10 s epoch with a frequency of 30 Hz. The PA data for each child was collected for 55 min during the GAP sesssion, and vector output was processed using ActiLife v6.15 software (ActiGraph, Pensacola, FL, USA) to estimate oxygen consumption (VO_2_) in mLO_2_·kg·min^−1^ and calculate PA energy expenditure (PAEE) in kcal·55 min^−1^ using a laboratory-generated linear regression equation for self-paced games [23]. For estimated VO_2_, the explained variance (R^2^) and standard error of estimate (SEE) using specific laboratory-based equations were 0.95 and 1.07 mLO_2_·kg·min^−1^, respectively [23]. Metabolic equivalents (MET) were estimated from laboratory-based equations [21,23] and were used to classify PA intensity as sedentary (≤1.9 METs), very light (2.0–2.9 METs), light (3.0–3.9 METs), moderate (4.0–5.9 METs), and vigorous (>6 METs). Intensity data is expressed in the percentage of time spent at moderate-to-vigorous PA (%MVPA), defined as >4 METs.

### 2.6. Statistical Analysis

Descriptive statistics, mean, and standard deviation for anthropometric, FMS, HRF, and PA variables were determined. Normality distribution for all variables was assessed by the skewness static divided by the standard error of skewness and/or the Shapiro–Wilk test. The prospective relationship analyses were conducted by multiple linear regression. Dependent variables consisted of PAEE and MVPA regressed with LOC, OC AP, LP, and STR as independent variables (as described in Stodden’s Hypothesis for children 8 to 10 years old). Bidirectional relationships were assessed with LOC and OC as dependent variables regressed for PAEE, MVPA, and components of HRF. Multiple linear regression results for unstandardized and/or standardized Beta (β) coefficients and the proportion of variation explained (R^2^) were used to evaluate relationships for PA, FMA, and HRF with SPSS v29. A post hoc power analysis was conducted using G*Power 3.1 to assess the adequacy of the sample size. With a moderate effect size f^2^ = 0.30, an alpha level = 0.05, *n* = 42, and 5 predictors, the calculated power was 0.93, indicating the study had sufficient power to detect moderate effects in the regression models

Categories for LOC, OC, LIA, OIA, and individual motors skills with outputs from guided active play cooperative games (PAEE and MVPA) and HRF components (AP, LP, and STR) were prepared using cut-off thresholds (percentiles) and classified into low (L) (<33%), medium M (33–66%), and high H (>66%) groups. Group means for FMS, PA, and HRF were compared with an analysis of variance and Tukey post-hoc mean differences. The associations between LOC, OC, LIA, and OIA with PA outputs and HRF classifications were assessed by a 3 × 3 chi-square Fisher’s exact test using the likelihood ratio for small cell sizes (<5). Cramer’s V statistic was used to determine the strength of a relationship between two categorical variables, classified as small (<0.15), medium (≥0.15 and <0.25), and large (≥0.25) [28]. Statistical comparisons of cross-tabulation analysis involved calculating z-scores from standardized residuals to determine alpha levels when comparing H and L classifications. The significance (*p* ≤ 0.05) levels were adjusted with the Bonferroni method to account for the number of statistical comparisons performed. All statistical analyses were performed with SPSS v29, and significance was set at *p* ≤ 0.05.

## 3. Results

### 3.1. Descriptive Results

Table 1 presents the mean anthropometric data, HRF measures, and FMS scores for boys and girls. The children in this study (mean age: 8.8 ± 0.8 years) had an average BMI of 18.9 ± 3.9 kg/m^2^ (Table 1A). According to the BMI reference chart for children, 5% of our children were underweight (<5th percentile), 57% were of healthy weight (>5th and <85th percentile), and 41% were overweight/obese (>85th percentile). Based on norm-referenced percentile values for grip strength, 85% of the girls and 86% of the boys fell within the 25th and 75th percentile range. For AP VO_2_max, 31% of the children were within the 25th and 75th percentile range, with girls being more predominant (40% of the girls compared to 23% of the boys). Regarding TGMD-2 percentiles, children were identified to be between the 25th and 37th percentile for LOC skills, OC skills, and the gross motor quotient (Table 1B). Based on gross motor quotient performance classifications, 28% of the children were categorized as very poor/poor, 33% as below average, 29% as average, and 10% as above average.

Guided active play (GAP) resulted in an average physical activity energy expenditure (PAEE) of 235.1 ± 74.8 kcal·55 min^−1^ and moderate-vigorous intensity (MVPA) of 38.5 ± 10.8%MVPA during the 55 min. PAEE for girls and boys was 219.6 ± 64.6 and 249.1 ± 82.0 kcal·55 min^−1^ (*p* > 0.05). MVPA for girls and boys was 32.5 ± 9.0 and 44.0 ± 9.4%MVPA (*p* ≤ 0.05).

### 3.2. Multiple Linear Regression Analysis for LOC and OC Skills with PAEE and MVPA and Components of HRF

Regression analyses showed that PAEE, MVPA, AP, LP, and STR were not significant predictors of LOC skill. However, MVPA significantly predicted OC skill (β = 0.394, *p* ≤ 0.01) (Table 2). The reverse pathway with LOC, OC, AP, LP, and STR as independent variables showed AP was a significant predictor of MVPA (β = 0.408, *p* ≤ 0.01), while LP was a significant predictor of PAEE (β = 0.456, *p* ≤ 0.01). LOC skill was not related to PAEE and MVPA during GAP (Table 2). In contrast, OC skill was related to MVPA (β = 0.308, *p* ≤ 0.05) but not PAEE. Similar results were observed for OIA, with MVPA (β = 0.498, *p* ≤ 0.01) but not for PAEE.

When adjusted for sex, MVPA was a significant predictor of LOC skills after a 1 h GAP session (β = 0.404, *p* ≤ 0.05). The PA outputs and components of HRF were not significant predictors of OC skill (Table 3). The reverse pathway with LOC, OC, AP, LP, and STR as independent variables showed AP was a significant predictor of MVPA (β = 0.309, *p* ≤ 0.05), while LP was a significant predictor of PAEE (β = 0.433, *p* ≤ 0.01) (Table 3).

Table 4 represents the relationship among FMS, PA outputs, and components of HRF during GAP, adjusted for BMI. Findings demonstrated that PAEE, MVPA, AP, LP, and STR were not significant predictors of LOC skill. However, MVPA significantly predicted OC skill (β = 0.395, *p* ≤ 0.05). The reverse pathway with LOC, OC, AP, LP, and STR as independent variables showed AP was a significant predictor of MVPA (β = 0.428, *p* ≤ 0.05). LOC skill did not predict PAEE and MVPA during GAP. In contrast, OC skill was related to MVPA (β = 0.305, *p* ≤ 0.05) but not PAEE.

### 3.3. Cut-Off Thresholds for LOC, OC, LIA, and OIA and Percentile Classifications for PA and HRF

Mean differences were observed between the low and high LOC, OC, LIA, and OIA classifications (*p* ≤ 0.05). Furthermore, the cut-off scores for the low and high classifications of all 12 individual motor skills were also statistically different (Supplementary Data—Table S1). Comparison of low and high classifications for the HRF components and PA outputs showed significant differences (*p* ≤ 0.05). Percentile classifications for GAP PAEE and MVPA and HRF components AP, LP, and STR are presented in Supplementary Table S2.

### 3.4. Association of Categories for LOC, OC, LIA, and OIA, Physical Activity and Health-Related Fitness Components

**Figure 1:**
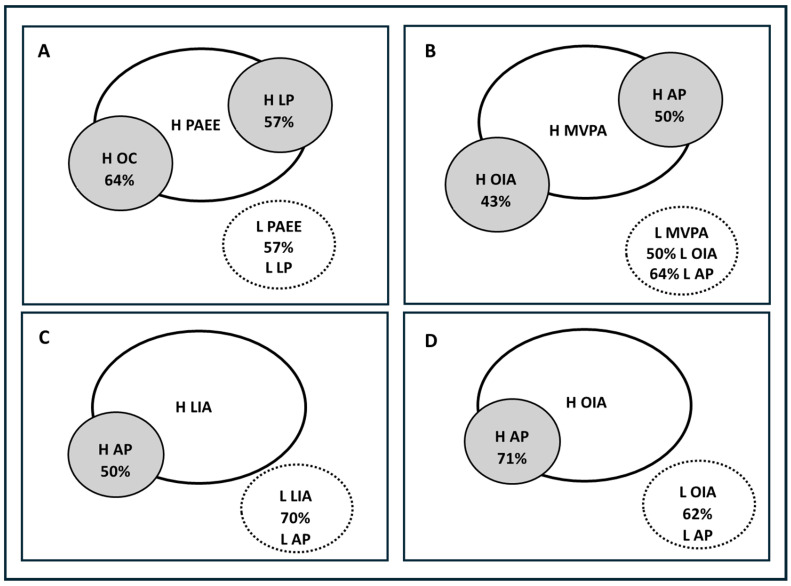
Schematic of chi-square and cross-tabulation results for the associations among fundamental movement skills, health-related fitness, and physical activity outputs from guided active play with cooperative games: (**A**) association of energy expenditure (PAEE) with OC and leg power (LP); (**B**) association of moderate-to-vigorous physical activity (MVPA) with individual average scores of OC skills (OIA) and aerobic power (AP); (**C**) association between individual average scores of LOC skills with AP; (**D**) association OIA and AP. Shaded ovals represent the percentage of children overrepresented within the high (H) category. Ovals with dotted lines represent the percentage of children overrepresented within the low (L) category. All cross-tabulations presented are statistically significant with *p* ≤ 0.05.

There was a significant association between PAEE and OC score (X^2^ = 15.12; df = 4; *p* ≤ 0.05; Cramer’s V = 0.44). Cross-tabulation showed that 64% of children with high OC performance were overrepresented in the high PAEE group (*p* ≤ 0.05). Considering LOC and OC scores, the application of cut-off thresholds showed non-significant associations with PAEE with LOC (X^2^ = 4.20; df = 4; *p* > 0.05) and MVPA (LOC X^2^ = 3.95; 4; *p* > 0.05; OC X^2^ = 5.23; 4; *p* > 0.05). A significant association was also found between OIA skills and MVPA but not with PAEE (X^2^ = 11.90; df = 4; *p* ≤ 0.05; Cramer’s V = 0.39). Cross-tabulation showed that 43% of children with high OIA scores were overrepresented in the high MVPA group (*p* ≤ 0.05). Conversely, 0% of children with high OIA were in the low MVPA group, whereas 50% of those with low OIA were overrepresented in the low MVPA group (*p* ≤ 0.05) (Figure 1). Additionally, the individual skill of striking was significantly associated with MVPA (X^2^ = 9.67; df = 4; *p* ≤ 0.05; Cramer’s V = 0.33). Regarding LIA, non-significant associations were observed with PAEE and MVPA.

Regarding HRF, PAEE showed a significant association with leg power (LP) (X^2^ = 11.88; df = 4; *p* ≤ 0.05; Cramer’s V = 0.38). Cross-tabulation revealed that 57% of children in the high LP category were overrepresented in the high PAEE group (*p* ≤ 0.05), while those in the low LP category (57%) were overrepresented in the low PAEE group (*p* ≤ 0.05). In contrast, non-significant associations were observed for LP with MVPA, LIA, and OIA. A significant association was also observed between estimated aerobic power (AP) and MVPA (X^2^ = 9.23; df = 4; *p* < 0.05; Cramer’s V = 0.33). There was an overrepresentation of children with high AP (43%) in the high MVPA group, and 64% of children with low AP were overrepresented in the low MVPA group (*p* ≤ 0.05). In addition, AP was also significantly associated with both LIA (X^2^ = 10.42; df = 4; *p* ≤ 0.05; Cramer’s V = 0.35) and OIA (X^2^ = 9.40; df = 4; *p* ≤ 0.05; Cramer’s V = 0.35). A total of 50% of children with high AP were overrepresented in the high LIA group, and 71% in the high OIA group. Conversely, 70% of children with low AP were overrepresented in the low LIA group, and 62% in the low OIA group (Figure 1). At the individual skill level, run, jump, slide, and dribble were each significantly associated with aerobic power. There was no significant categorical association of strength with PA outputs and FMS during guided active play.

## 4. Discussion

This study explored the relationships among fundamental motor skills (FMS), health-related fitness (HRF) variables, and physical activity (PA) outputs from a 1 h guided active play (GAP) session. In general, we observed that older children’s participation in GAP was positive with an average PA energy expenditure (PAEE) of ~240 kcal·55 min^−1^ and intensity of 38.5% MVPA, or ~28 min. This agrees with our previous reports of high enjoyment levels for GAP [21]. Standardized regression weights showed a positive reciprocal effect of object control skills (OC) on MVPA, and MVPA on OC for GAP, which aligns with Stodden’s hypothesis of integrated developmental trajectories for older children [5]. Moreover, aerobic power (AP) significantly predicted MVPA, such that 1 SD change in AP increased MVPA by ~ 10% MVPA. The observation that leg power (LP) predicted PAEE for GAP implicates muscle fitness as an important component of children’s fitness. When adjusted for body mass index, the reciprocal relationships between OC and MVPA and AP on MVPA remained; however, the impact of LP on PAEE was not significant. Sex adjustments showed a reduced impact for OC on MVPA, and MVPA on OC, albeit the AP and LP effects on MVPA and PAEE, respectively, remained. The observation that significant associations exist between high individual average scores of OC (OIA) and MVPA agreed with the standardized coefficients observed for OC and MVPA. In summary, the similarity in findings among FMS, HRF, and PA variables for GAP are reassuring and suggestive of strong relationships/associations whether analyzed by predicting variables (regression) or mean comparisons (chi-square/ANOVA). Therefore, our GAP results showing strong relationships between FMS and PA support the call for active play initiatives to promote childhood health issues through play-based physical activity promotion [29,30].

A systematic review and meta-analysis concluded that time in MVPA predicts OC levels, and OC levels predict MVPA, but not LOC for children 3–6 years old [31]. Our results extend the positive reciprocal relationships between OC levels and MVPA to older children for GAP. The GAP results do not agree with a report on children during middle childhood showing non-significant indirect effects between school-based MVPA and FMS [8]. The school-based results are not entirely unexpected, since the school-based PA was assessed and accumulated over several days with different types and domains of PA, possibly leading to biased relationships with FMS [11,12]. Results of the current study revealed a positive reciprocal bidirectional relationship between MVPA and OC when PA is collected for a 1 h GAP format that provides fun, self-paced, self-chosen cooperative games [21,32]. Furthermore, accelerometer outputs were used to estimate metabolically related PAEE (kcal·55 min^−1^) and MVPA using METs rather than motion-based vectors for determining intensity by vector cut-offs [21,23]. These results support the use of selected movement-based PA domains when assessing relationships with FMS and HRF for performance- and health-related outcomes.

Observations that the classifications for OIA and LIA ranged from proficiency to mastery levels supported the use of high cut-off scores to provide relevant categories to assess the association between FMS and PA. Children with high OIA scores and high MVPA showed significant associations and agreed with the analysis using multiple linear regression. Together our findings support the positive reciprocal relationship between OC and MVPA during middle and late childhood proposed in the developmental trajectory model [5]. Interestingly, the categorical results obtained for GAP identify the proficiency level for OIA, which is associated with high levels of MVPA. This association has been suggested to be important for PA participation in sports [33,34] and supports Seefeldt’s ‘proficiency barrier’ linking children’s FMS proficiency levels to PA [35,36].

Previous research has shown a strong positive correlation between FMS proficiency and individual components of HRF components in children and adolescents [3,37,38,39]. While a longitudinal study has revealed a bidirectional predictive effect of physical fitness with LOC and OC skills in early childhood [40], such relationships have not been observed for the middle to late childhood stage. In this current study, regression models showed that HRF components did not correlate with FMS proficiency when adjusted or not adjusted for BMI during middle to late childhood (8 to 10-year-old children). However, categorical analyses showed high classifications of LIA and OIA were significantly associated with the high categories for AP. This supports the approach of using a cut-off threshold when computing sum and individual average scores of TGMD-2 outputs to ob-serve significant relationships between FMS and HRF components [19]. Strength and leg power was not associated with FMS proficiency in this developmental stage. Additionally, few studies have shown the direct response of PA on HRF variables in middle to late childhood. Results showed a strong predictive effect of AP on MVPA when adjusted for sex and BMI separately. LP predicted PAEE when only sex was adjusted for. Analyses utilizing cut-off thresholds confirmed a significant strong association of high performance of MVPA and PAEE with high AP and LP. These findings suggest a robust relationship be-tween MVPA and AP (without the influence of sex and BMI) and PAEE and LP (without the influence of sex). The association of AP with MVPA from GAP, LIA, and OIA aligns with prior research that suggests an association between FMS proficiency and AP that strengthens with age during childhood [3,38,41]. Strength did not correlate with FMS proficiency and PA outputs for 8 to 10-year-old children. Future studies will consider resistance training interventions that will improve HRF variables, including muscle strength, power, and endurance [42]. Such interventions may help to promote the association between FMS and HRF, ultimately leading to improved health outcomes for children.

To date, no study has investigated the influence of sex in the association of PA during GAP and FMS in middle to late childhood. In this study, regression analyses without sex adjustment revealed a strong bidirectional predictive relationship between MVPA and OC skills. Secondary analysis of categorical associations showed that a high level of PAEE was significantly associated with high OC, particularly for boys, but not for girls. This suggests that for boys 8 to 10 years, OC proficiency will improve MVPA and PAEE from 1-h GAP and vice versa. This finding agrees with a study that showed an association between OC skill, specifically catch, for boys with habitual PA for children across childhood [43]. While the observed sex-specific differences in the association between PA and FMS for 8 to 10-year-old children are novel, the limited sample size and statistical power restrict the generalizability of these findings. Future research with larger samples is needed to investigate these sex-related differences further and strengthen the understanding of FMS and GAP PA interactions in childhood development.

Additional limitations include the logistical constraint(s) of working in community settings, which can pose challenges such as recruitment difficulties and high attrition rates. Children in these settings may face life circumstances that prevent them from com-pleting some parts of the assessment process, thus reducing the final sample size. This reduction in sample size influences the generalizability of the results; however, our ob-servations that the children’s distribution of gross motor quotient performance categories [27,44,45], as well as children’s PA participation levels [16,23] were within published requirements. Furthermore, the distribution of children’s AP and LP, showed that our children were relatively distributed with no one factor being dominant. 

Finally, there is limited understanding of the levels of LOC and OC utilized during GAP with cooperative games. Enhancing our understanding of guided active play is important when designing PA programs tailored to improving specific FMS. Without this knowledge of the extent to which these skills are engaged, it becomes difficult to develop targeted interventions that effectively improve FMS proficiency and HRF outcomes. Future studies should focus on the effect of individual sport-related games that consider LOC and OC levels in the association of PA with FMS proficiency and HRF.

## 5. Conclusions

This study confirms that GAP is a viable option for children’s PA that shows a positive reciprocal relationship between OC and MVPA for 8- to 10-year-old children. The role of HRF, in particular AP, and LP to a lesser extent, is also highlighted with GAP. These results were also verified by using cut-off thresholds FMS scores compared to PA and HRF variables. Together, these approaches can help identify key focus areas for PA programming aimed at improving motor skill development and fitness levels in children. The application of metabolically determined PAEE and MVPA also provides valuable insights for educators and researchers. Such approaches may enhance understanding of FMS development and proficiency, as well as the impact of fitness on PA outputs during the critical developmental stage of middle to late childhood.

## Figures and Tables

**Table 1:** Children’s physical characteristics and health-related fitness (HRF) (**A**) are presented for body mass (BM), height, body mass index (BMI), average strength (STR), leg power (LP), and aerobic power (AP). Test of Gross Motor Development-2 scores are provided for locomotor (LOC) skills, object control (OC) skills, and gross motor quotient (GMQ) (**B**) for summed, standard, and percentiles. Statistical comparisons for OC skills showed boys higher than girls (* at *p* ≤ 0.05).

A	Anthropometric Variables					Health-Related Fitness		
	Age (yrs)	BM (kg)	Height (cm)	BMI (kg·m^2^)		STR (kg)	LP (W)	AP (mLO_2_,· kg·min^−1^)
Total (*n* = 42)	8.8 ± 0.8	36.4 ± 12.3	136.9 ± 9.8	18.9 ± 3.9		27.5 ± 4.8	788.0 ± 409.4	43.8 ± 4.8
Girls (*n* = 20)	8.9 ± 0.9	37.0 ± 10.8	137.6 ± 9.4	19.2 ± 3.7		28.1 ± 6.1	774.2 ± 450.8	41.6 ± 3.8
Boys (*n* = 22)	8.7 ± 0.8	35.8 ± 13.7	136.2 ± 10.4	187 ± 4.2		26.9 ± 5.9	800.6 ± 378.1	45.7 * ± 4.9
**B**	**Sum Scores**		**Standard Scores**			**Percentile Classification**		
	**LOC**	**OC**	**LOC**	**OC**	**GMQ**	**LOC**	**OC**	**GMQ**
Total (*n* = 42)	37.9 ± 8.2	38.6 ± 6.3	7.6 ± 3.1	8.5 ± 2.8	86.9 ± 15.5	30.3 ± 28.6	32.7 ± 26.2	26.2 ± 25.1
Girls (*n* = 20)	38.6 ± 6.7	35.1 ± 5.4	7.6 ± 2.7	8.2 ± 2.8	85.3 ± 15.5	31.2 ± 29.4	27.8 ± 26.5	24.8 ± 27.4
Boys (*n* = 22)	37.2 ± 9.4	41.8 * ± 5.4	7.6 ± 3.5	8.7 ± 2.9	88.3 ± 15.7	29.5 ± 28.6	37.2 ± 25.6	27.5 ± 23.5

**Table 2:** Summary of multiple linear regression parameter estimates for locomotor (LOC) and object control (OC) skills with physical activity during guided active play and health-related fitness not adjusted for sex and body mass index.

	Independent Variables				
Dependent Variables	PAEE β CI95% (L, H)	MVPA β CI95% (L, H)	AP β CI95% (L, H)	LP β CI95% (L, H)	STR β CI95% (L, H)
LOC F 1.39, df 5, R^2^ = 0.16 *p* > 0.05	−0.259 (−0.067, 0.011) *p* > 0.05	0.262 (−0.101, 0.497) *p* > 0.05	0.083 (−0.547, 0.829) *p* > 0.05	0.195 (−0.004, 0.011) *p* > 0.05	0.006 (−0.501, 0.518) *p* > 0.05
OC F 3.39, df 5, R^2^ = 0.32 *p* ≤ 0.05	−0.037 (−0.030, 0.024) *p* > 0.05	0.394 (0.022, 0.438) *p* ≤ 0.05	0.259 (−0.140, 0.817) *p* > 0.05	0.027 (−0.005, 0.006) *p* > 0.05	0.081 (−0.269, 0.440) *p* > 0.05
	**LOC**	**OC**	**AP**	**LP**	**STR**
PAEE F 2.81, df 5, R^2^ = 0.28 *p* ≤ 0.05	−0.250 (−5.419, 0.831) *p* > 0.05	0.149 (−2.637, 6.167) *p* > 0.05	−0.131 (−7.417, 3.347) *p* > 0.05	0.456 (0.025, 0.142) *p* ≤ 0.01	0.024 (−3.984, 4.597) *p* > 0.05
MVPA F 5.81, df 5, R^2^ = 0.45 *p* ≤ 0.001	0.013 (−0.378, 0.413) *p* > 0.05	0.308 (0.063, 1.083) *p* ≤ 0.05	0.408 (0.232, 1.593) *p* ≤ 0.01	0.145 (−0.004, 0.011) *p* > 0.05	−0.130 (−0.777, 0.308) *p* > 0.05

Note: β, beta; CI95%, confidence interval for 95%; L, lower; U, upper; PAEE, energy expenditure; MVPA, percent time spent in moderate-to-vigorous PA; AP, aerobic power, LP, Leg power; STR, average grip strength; *p* refers to alpha level.

**Table 3:** Summary of multiple linear regression parameter estimates for locomotor (LOC) and object control (OC) skills with physical activity during guided active play and health-related fitness with sex adjustments with girls as the reference category.

	Independent Variables					
Dependent Variables	PAEE β CI95% (L, H)	MVPA β CI95% (L, H)	AP β CI95% (L, H)	LP β CI95% (L, H)	STR β CI95% (L, H)	Sex β CI95% (L, H)
LOC F 1.84, df 6, R^2^ = 0.24 *p* > 0.05	−0.169 (−0.058, 0.021) *p* > 0.05	0.404 (0.006, 0.617) *p* ≤ 0.05	0.173 (−0.392, 0.977) *p* > 0.05	0.152 (−0.004, 0.010) *p* > 0.05	0.037 (−0.445, 0.545) *p* > 0.05	−0.350 (−11.973, 0.419) *p* > 0.05
OC F = 3.95, df 6, R^2^ = 0.40 *p* ≤ 0.01	−0.131 (−0.038, 0.016) *p* > 0.05	0.246 (−0.069, 0.357) *p* > 0.05	0.166 (−0.251, 0.686) *p* > 0.05	0.071 (−0.004, 0.006) *p* > 0.05	0.049 (−0.286, 0.390) *p* > 0.05	0.364 (0.382, 8.691) *p* ≤ 0.05
	**LOC**	**OC**	**AP**	**LP**	**STR**	**Sex**
PAEE F = 2.78, df 6, R^2^ = 0.32 *p* ≤ 0.05	−0.086 (−4.497, 2.927) *p* > 0.05	−0.067 (−6.385, 4.790) *p* > 0.05	−0.206 (−8.736, 2.339) *p* > 0.05	0.433 (0.021, 0.137) *p* ≤ 0.01	0.022 (−3.945, 4.508) *p* > 0.05	0.303 (−1.827, 6.520) *p* > 0.05
MVPA F = 6.32, df 6, R^2^ = 0.52 *p* ≤ 0.001	0.231 (−0.145, 0.756) *p* > 0.05	0.022 (−0.640, 0.716) *p* > 0.05	0.309 (0.019, 1.363) *p* ≤ 0.05	0.116 (−0.004, 0.010) *p* > 0.05	-0.132 (−0.752, 0.274) *p* > 0.05	0.401 (1.063, 6.031) *p* ≤ 0.05

Note: β, beta; CI95%, confidence interval for 95%; L, lower; U, upper; PAEE, energy expenditure; MVPA, percent time spent in moderate-to-vigorous PA; AP, aerobic power; LP, Leg power; STR, average grip strength. *p* refers to alpha level.

**Table 4:** Summary of multiple linear regression parameter estimates for locomotor (LOC) and object control (OC) skills with physical activity during guided active play and health-related fitness with body mass index adjustments.

	Independent Variables					
Dependent Variables	PAEE β CI95% (L, H)	MVPA β CI95% (L, H)	AP β CI95% (L, H)	LP β CI95% (L, H)	STR β CI95% (L, H)	BMI β CI95% (L, H)
LOC F 1.149, df 6, R^2^ = 0.17 *p* > 0.05	−0.317 (−0.088, 0.019) *p* > 0.05	0.267 (−0.101, 0506) *p* > 0.05	0.112 (−0.563, 0.942) *p* > 0.05	0.173 (−0.005, 0.012) *p* > 0.05	−0.008 (−0.538, 0.517) *p* > 0.05	0.140 (−1.030, 1.460) *p* > 0.05
OC F 2.75, df 6, R^2^ = 0.32 *p* ≤ 0.05	−0.055 (−0.042, 0.033) *p* > 0.05	0.395 (0.020, 0.443) *p* ≤ 0.05	0.268 (−0.174, 0.874) *p* > 0.05	0.020 (−0.005, 0.006) *p* > 0.05	0.077 (−0.286, 0.449) *p* > 0.05	0.032 (−0.817, 0.918) *p* ≤ 0.05
	**LOC**	**OC**	**AP**	**LP**	**STR**	**BMI**
PAEE F 8.87, df 6, R^2^ = 0.60 *p* ≤ 0.001	−0.165 (−3.885, 0.866) *p* > 0.05	0.101 (−2.132, 4.520) *p* > 0.05	0.173 (−1.751, 7.127) *p* > 0.05	0.90 (−0.035, 0.068) *p* > 0.05	−0.099 (−4.525, 2.052) *p* > 0.05	0.810 (9.487, 21.150) *p* ≤ 0.001
MVPA F 4.73, df 6, R^2^ = 0.45 *p* ≤ 0.001	0.019 (−0.397, 0.429) *p* > 0.05	0.305 (0.044, 1.087) *p* ≤ 0.05	0.428 (0.203, 1.712) *p* ≤ 0.05	0.121 (−0.004, 0.012) *p* > 0.05	−0.138 (−0.752, 0.310) *p* > 0.05	0.054 (−0.846, 1.137) *p* > 0.05

Note: β, beta; CI95%, confidence interval for 95%; L, lower; U, upper; PAEE, energy expenditure; MVPA, percent time spent in moderate-to-vigorous PA; AP, aerobic power, LP, Leg power; STR, average grip strength; BMI; body mass index; *p* refers to alpha level.

## Data Availability

The datasets generated during and/or analyzed during the current study are not publicly available but are available from the corresponding author, who was an organizer of the study.

## Supplementary Materials

The following Supporting Information can be downloaded at: https://www.mdpi.com/article/10.3390/children12060805/s1, Table S1: Cut-Off Thresholds for Fundamental Movement Skills; Table S2: Cut-Off Thresholds for Physical Activity and Health-related Fitness. Figure S1: Path Analysis Relationships with Path Coefficients between Physical Activity (PA) and Fundamental Movement Skills. Figure S2: Path Analysis Relationships with Path Coefficients between Fundamental Movement Skills and Physical Activity.
