# Supplementary material for: The Role of Fundamental Movement Skills and Health-Related Fitness on Physical Activity During Guided Active Play for 8- to 10-Year-Old Children
Source: Children (Basel). 2025 Jun 19;12(6):805. doi: 10.3390/children12060805 (PMC12192467; doi:10.3390/children12060805)
Supplement: Supplementary file 1 [file children-12-00805-s001.zip › children-3642137-supplementary.pdf]

Gross Motor Competence and Active Play - Supplementary Data – Path Analysis Figure S1 and Figure S2

A.

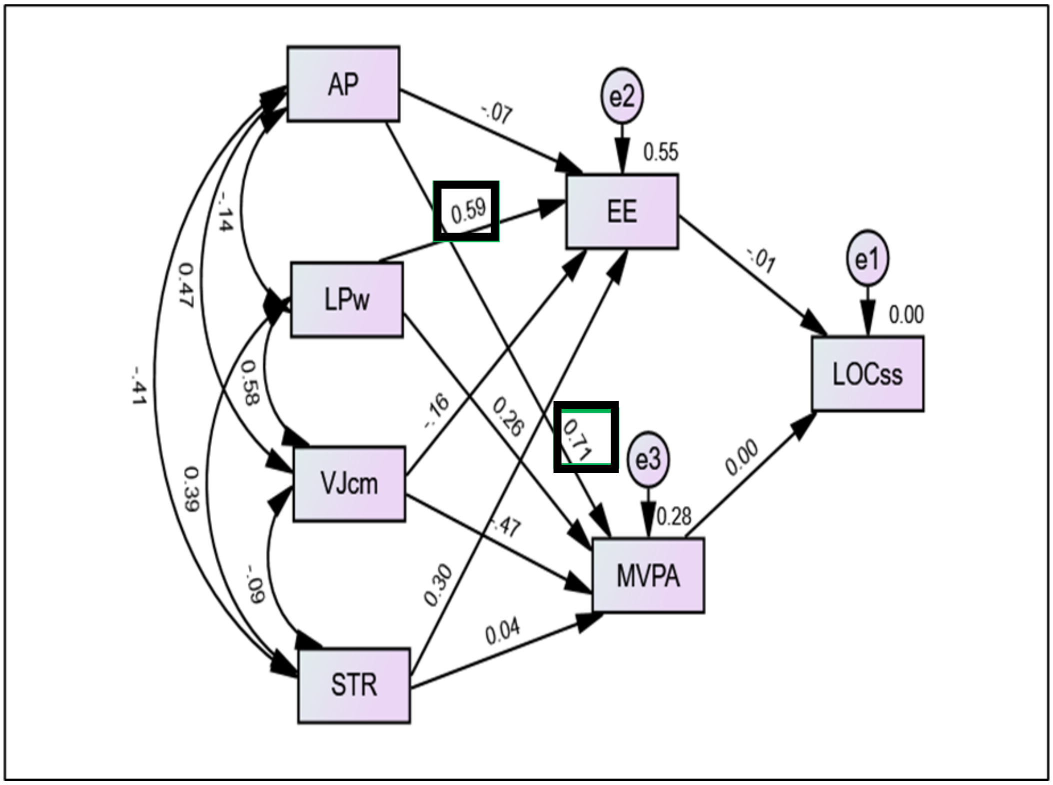

B.

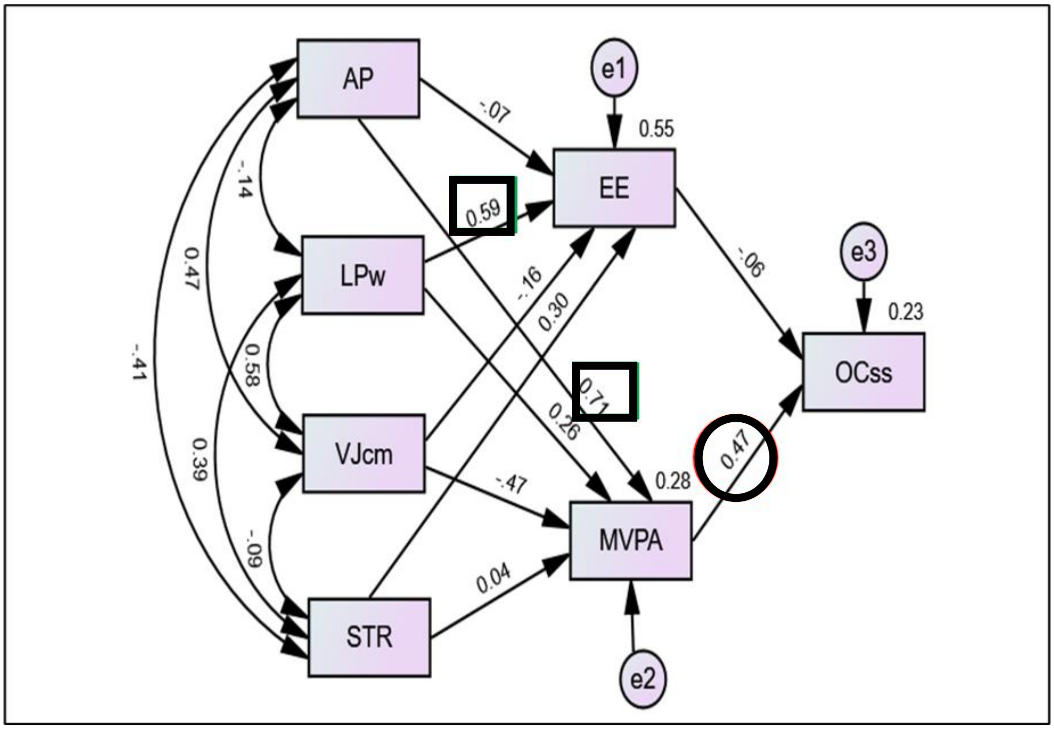

Figure S1: Path Analysis Relationships with Path Coefficients between Physical Activity (PA) and Fundamental Movement Skills. Key variables include locomotor skill sum score (LOCss), object control skill sum score (OCss), physical activity outputs for energy expenditure (EE), and the percent of time spent at moderate-to-vigorous physical activity (%MVPA). A) PAEE and MVPA → LOC (model 1), B) PAEE and MVPA → OC (model 2). Health-related Fitness (HRF) components, including aerobic power (AP), leg power (LPw), vertical jump (VJcm), and strength (STR), are included. Rectangles = indicators; ovals = unobserved variance (e); single arrow = direct effect specified by β coefficient; double-headed arrows = covariances or correlations.; bolded circles = significant direct effect; bolded square = significant covariate analysis for HRF, $p \leq 0.05$.

A.

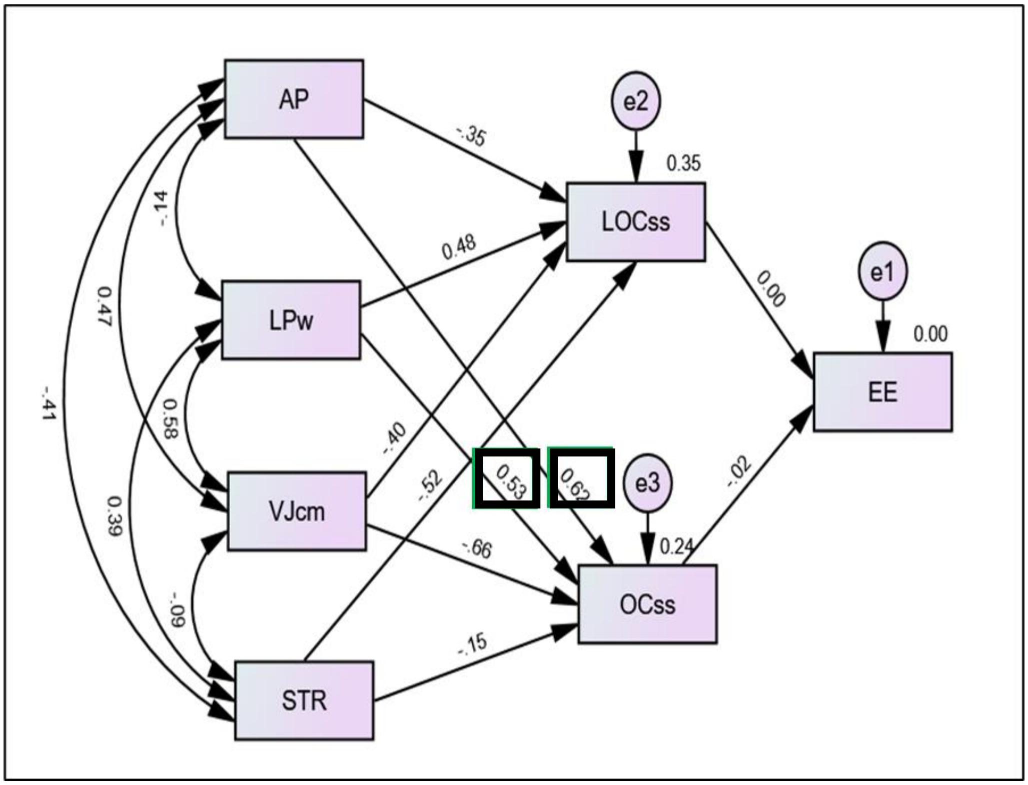
AP
LPw
VJcm
STR
LOCss
OCss
EE
e1
e2
e3
0.35
0.00
0.24
-.35
0.48
-.40
-.52
0.53
0.62
-.66
-.15
0.00
-.02
-.14
0.47
0.58
-.41
0.39
-.09

B.

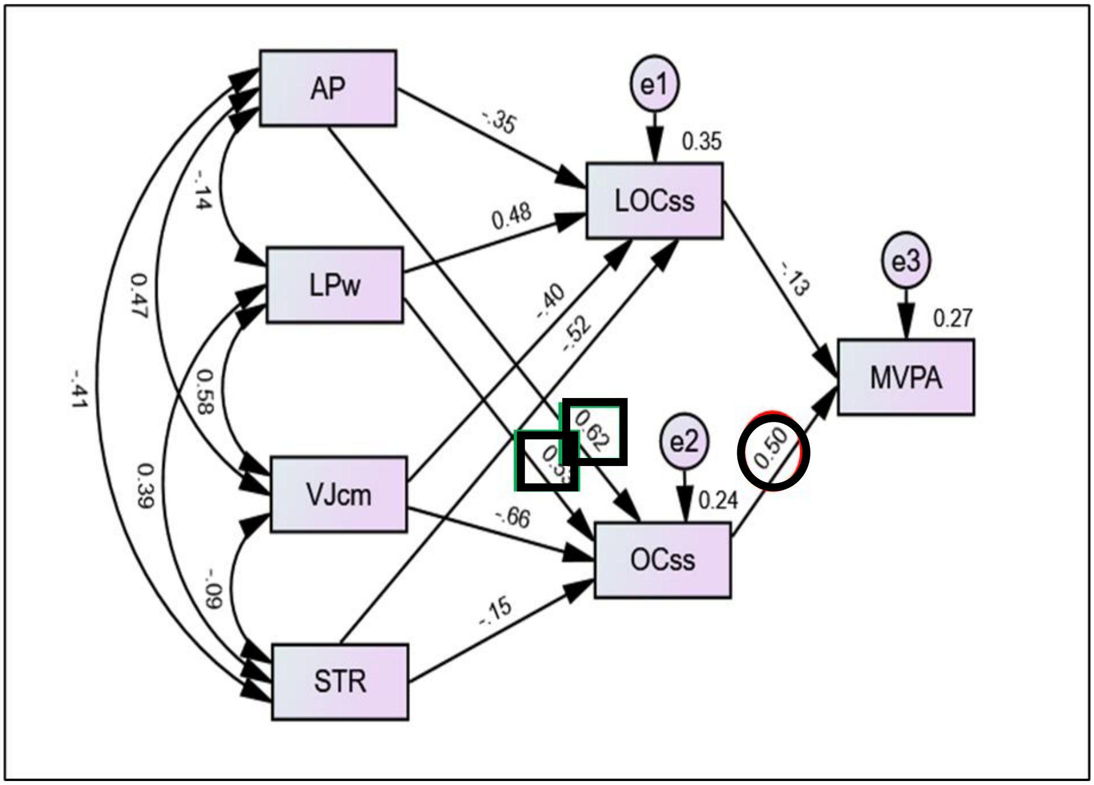
AP
LPw
VJcm
STR
LOCss
OCss
MVPA
e1
e2
e3
0.35
0.27
0.24
-.35
0.48
-.40
-.52
0.62
-.66
-.15
-.13
0.50
-.14
0.47
0.58
-.41
0.39
-.09

Figure S2: Path Analysis Relationships with Path Coefficients between Fundamental Movement Skills and Physical Activity. Key variables include locomotor skill sum scores (LOCss), object control skill sum scores (OCss), physical activity outputs for energy expenditure (EE), and the percent of time spent at moderate-to-vigorous physical activity (%MVPA). A) LOC and OC → PAEE (model 3), and B) LOC and OC → MVPA (model 4). Health-related Fitness (HRF) components, including aerobic power (AP), leg power (LPw), vertical jump (VJcm), and strength (STR), are included. Rectangles = indicators; ovals = unobserved variance (e); single arrow = direct effect specified by β coefficient; double-headed arrows = covariances or correlations; bolded circle = significant direct effect; bolded square = significant covariate analysis for HRF, $p \leq 0.05$.

Gross Motor Competence and Active Play - Supplementary Data – Classifications for Fundamental Movement Skills - Table S1 and Physical Activity and Health Related Fitness – Table S2

Table S1: Children's Low (<33%) and High (>66%) Classifications for Locomotor (LOC), Object Control (OC), Individual Averaged Locomotor (LIA) and Object Control (OIA) Skills Were Separated into Age, Sex (G-Girl; B-Boy), and the Number (#) and Frequency (%) of Children. Each of the 12 Individual Motor Skill Scores Were Classified Accordingly. Statistical Comparisons Between Low and High Classifications for LOC, OC LIA, OIA, and Individual Scores Are Identified with * p≤0.05. p = proficiency; m = mastery.

Variable	Low (<33%)	Freq # (%)	Age (yrs)	Sex	High (>66%)	Freq # (%)	Age (yrs)	Sex
**LOC and OC Scores - Laboratory Percentiles (n=21)**								
**LOC**	≤ 38	7 (33)	8.7	G2 B5	≥ 44*p	6 (29)	8.2	G2 B4
**OC**	≤ 37	6 (29)	8.5	G4 B2	≥ 44*p	9 (43)	8.7	G2 B7
**LOC and OC Individual Averaged Scores-Laboratory Percentiles (n=21)**								
**LIA**	≤ 6	7 (33)	8.7	G2 B7	≥ 8*, p	5 (24)	8.2	G1 B4
**OIA**	≤ 6	6 (29)	8.5	G4 B2	≥ 8*, p	5 (24)	8.2	G0 B5
**Individual Skill Scores-Laboratory Percentiles (n=21)**								
**Run**	≤ 6	12 (57)	8.5	G5 B7	≥ 8*, m	6 (29)	8.5	G3 B3
**Gallop**	≤ 6	8 (38)	8.6	G3 B5	≥ 8*, m	11 (52)	8.5	G4 B7
**Hop**	≤ 7	5 (24)	8.8	G2 B3	≥ 9*, p	7 (33)	8.3	G2 B5
**Leap**	≤ 4	7 (33)	8.8	G4 B3	≥ 6*, m	11(53)	8.3	G2 B9
**Jump**	≤ 6	8 (38)	8.9	G3 B5	≥ 8*, m	6 (29)	8.2	G1 B5
**Slide**	≤ 6	2 (9)	9.0	G0 B2	≥ 8* m	19 (90)	8.5	G8 B11
**Strike**	≤ 6	7 (33)	8.4	G5 B2	≥ 10*m	7 (33)	8.1	G0 B7
**Dribble**	≤ 6	5 (24)	8.6	G2 B3	≥ 8*m	9 (43)	8.4	G1 B8
**Catch**	≤ 5	6 (29)	8.5	G2 B4	≥ 6*, m	15 (71)	8.5	G6 B9
**Kick**	≤ 6	7 (33)	8.6	G4 B3	≥ 8*, m	13 (62)	8.5	G3 B10
**Throw**	≤ 6	10 (40)	8.7	G4 B6	≥ 8*m	8 (30)	8.5	G3 B5
**Roll**	≤ 4	5 (24)	8.6	G3 B2	≥ 8*m	11 (52)	8.6	G3 B8

Table S2: Health-Related Fitness (HRF) Components of Aerobic Power (AP Estimated VO_2max), Leg Power (LP), And Average Grip Strength (STR) Were Classified into Low and High Categories. Physical Activity Outputs from Guided Active Play Sessions Were Partitioned into Low and High Percentiles For Energy Expenditure (PAEE) ($kcal \cdot 55min^{-1}$) and Percent Time at Moderate-Vigorous Physical Activity (%MVPA). The Number of Children in Each Classification (Freq) and the Number of Girls (G) and Boys (B) Are Included. Significant Mean Comparisons (*) Between the Variable Low and High Categories Were Set at a $p \leq 0.05$.

Variable	Low (<33%)	Freq # (%)	Age (yrs)	Sex	High (>66%)	Freq # (%)	Age (yrs)	Sex
**Physical Activity Outputs (n=21)**								
**PAEE (kcal.55min-1)**	≤ 196.0	7 (33)	8.3	G3 B4	≥ 292.2*	7 (33)	8.4	G2 B5
**MVPA (%)**	≤ 38.9	6 (29)	8.3	G5 B1	≥ 44.9*	8 (38)	8.5	G1 B7
**Components of Health-related Fitness (n=21)**								
**AP ($mlO_2 \cdot kg \cdot min^{-1}$)**	≤ 44.1	8 (38)	8.6	G4 B4	≥ 48.6*	7 (33)	8.6	G1 B6
**LP (W)**	≤ 517.0	7 (33)	8.1	G3 B4	≥ 935.6*	7 (33)	8.6	G4 B3
**STR (kg)**	≤ 22.1	7 (33)	8.6	G1 B6	≥ 25.9*	7 (33)	8.9	G3 B4
